# Supplementary material for: Nucleolar reorganization after cellular stress is orchestrated by SMN shuttling between nuclear compartments
Source: Nat Commun. 2023 Nov 15;14:7384. doi: 10.1038/s41467-023-42390-4 (PMC10652021; doi:10.1038/s41467-023-42390-4)
Supplement: Supplementary file 1 — Supplementary Information [file 41467_2023_42390_MOESM1_ESM.pdf]

NUCLEOLAR REORGANIZATION AFTER CELLULAR STRESS IS ORCHESTRATED BY SMN SHUTTLING BETWEEN NUCLEAR COMPARTMENTS

SUPPLEMENTARY INFORMATION

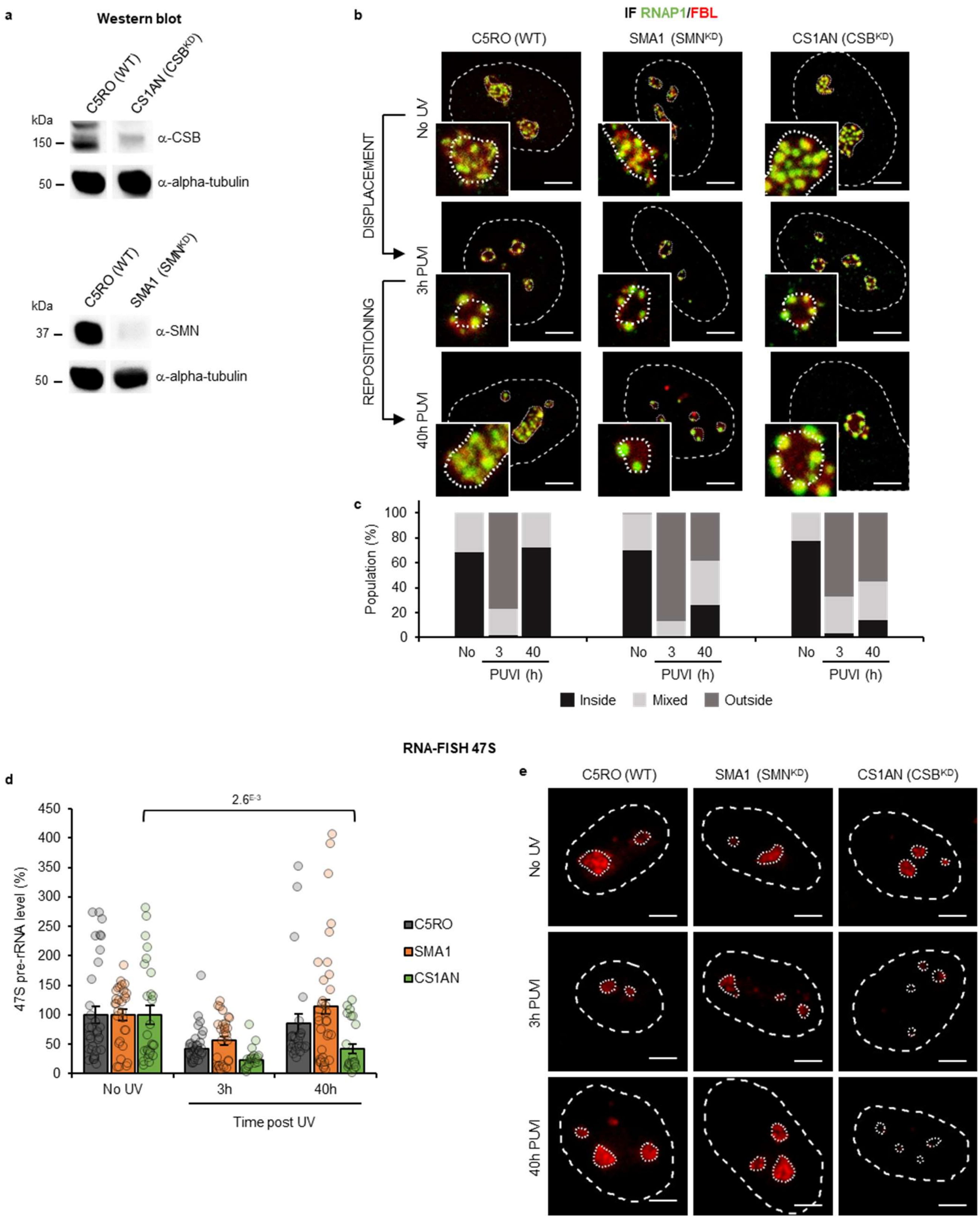

Figure S1: RNAP1 and FBL localization during DNA repair in SMN deficient transformed fibroblasts.

a. Western Blot of SMN and CSB on whole cell extracts of primary fibroblasts. **b.** Representative confocal microscopy images of immunofluorescence (IF) assay against RNAP1 (green) and FBL (red) in primary fibroblasts, at different times

Post UV-Irradiation (PUVI). Nuclei and nucleoli are indicated by dotted lines. Scale bar: 5µm**.** **c.** Quantification of cell number for RNAP1 localization (inside the nucleolus, outside the nucleolus or mixed localization) at different times PUVI. At least 30 cells from one representative experiment were analyzed. **d.** Quantification of RNA-FISH assay showing the 47S pre-rRNA level after UV-C exposure in primary fibroblasts. Data are represented as mean values +/- SEM. At least 18 cells was quantified from one representative experiment. The p-value correspond to a student's test with two-tailed distribution and two-sample unequal variance to compare after irradiation with No UV condition. **e.** Representative images of RNA-FISH 47S in primary fibroblasts from Fig. S1d. Nuclei and nucleoli are indicated by dotted lines. Scale bar 5µm

Source data of uncropped gel and graphs are provided as a Source Data file.

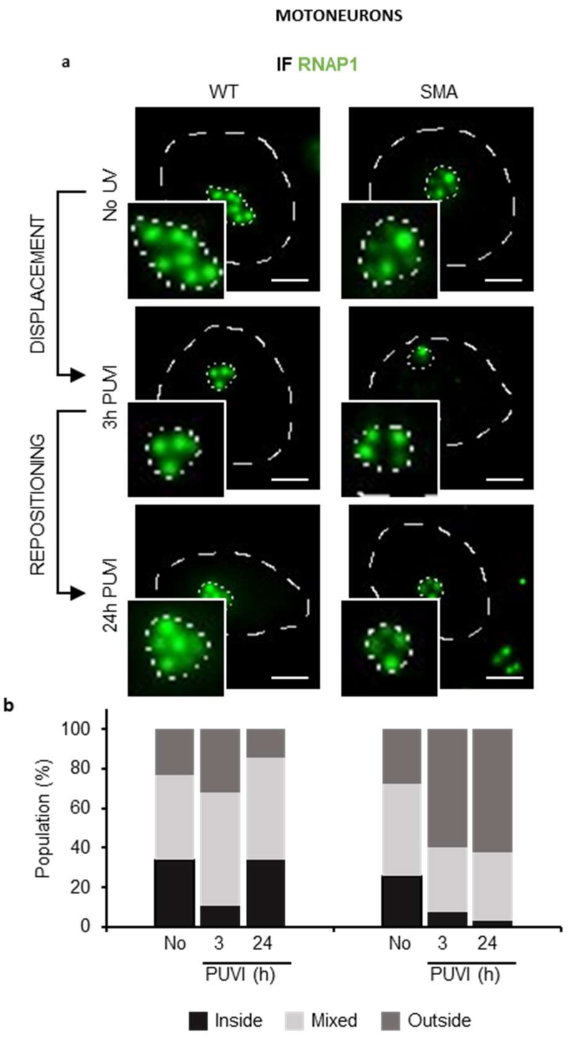

Figure S2: RNAP1 localization during DNA repair in iPSC-derived motoneurons.

a. Representative microscopy images of immunofluorescence (IF) assay against RNAP1 (green) in iPSC-derived motoneurons from control (WT) or SMA patients, at different times Post UV-Irradiation (PUVI). Nuclei and nucleoli are indicated by dotted lines. Scale bar: 5µm. **b.** Quantification of the number of cells for RNAP1 localization (inside the nucleolus, outside the nucleolus or mixed localization) at different times PUVI. At least 32 cells from one representative experiment were analyzed.

Source data of graphs are provided as a Source Data file.

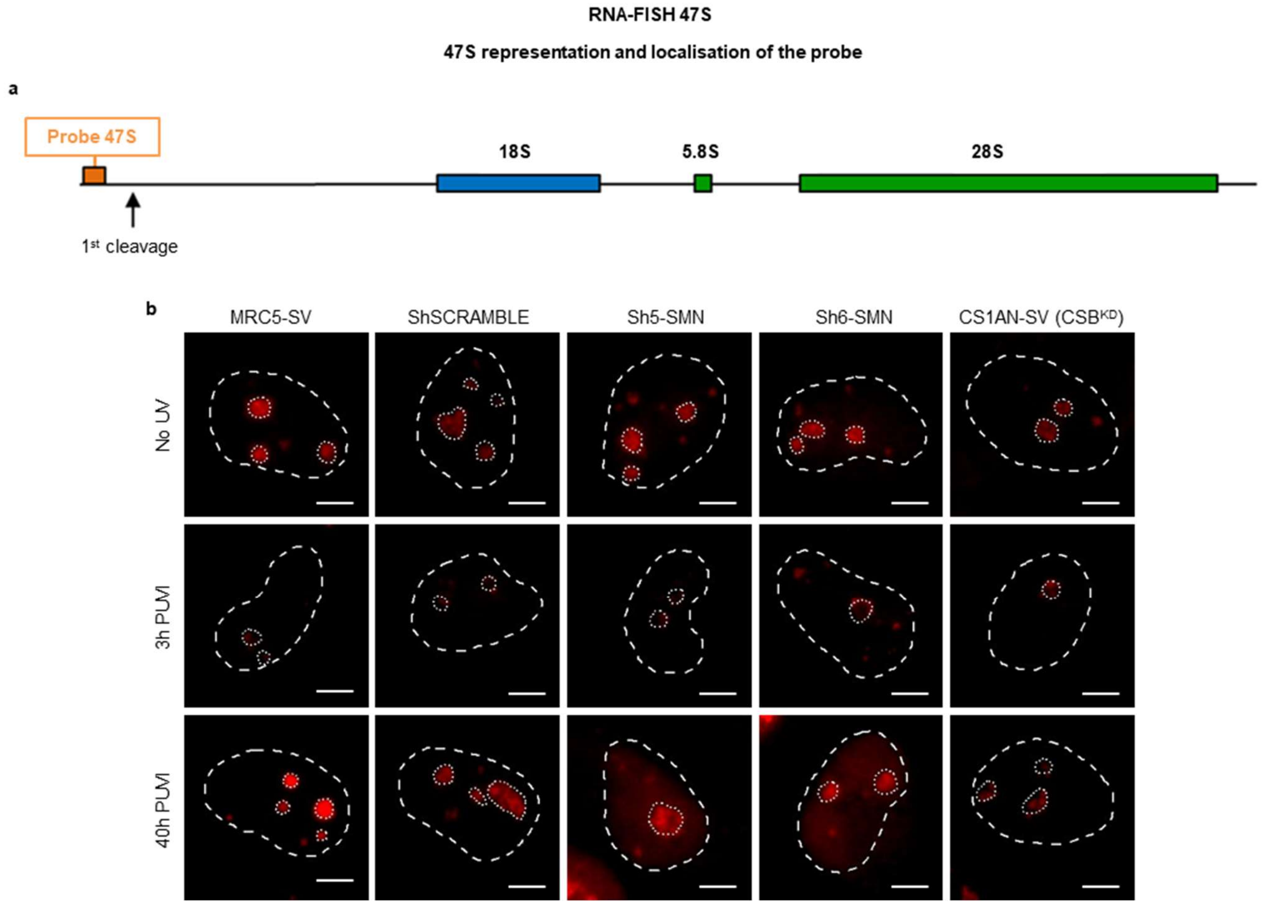

Figure S3: RNA-FISH 47S in transformed fibroblasts.

a. Schematic representation of rRNA unit and localization of the 47S pre-RNA probe. **b.** Representative images of RNA-FISH 47S in transformed fibroblasts from Fig. 1d. Nuclei and nucleoli are indicated by dotted lines. Scale bar: 5µm.

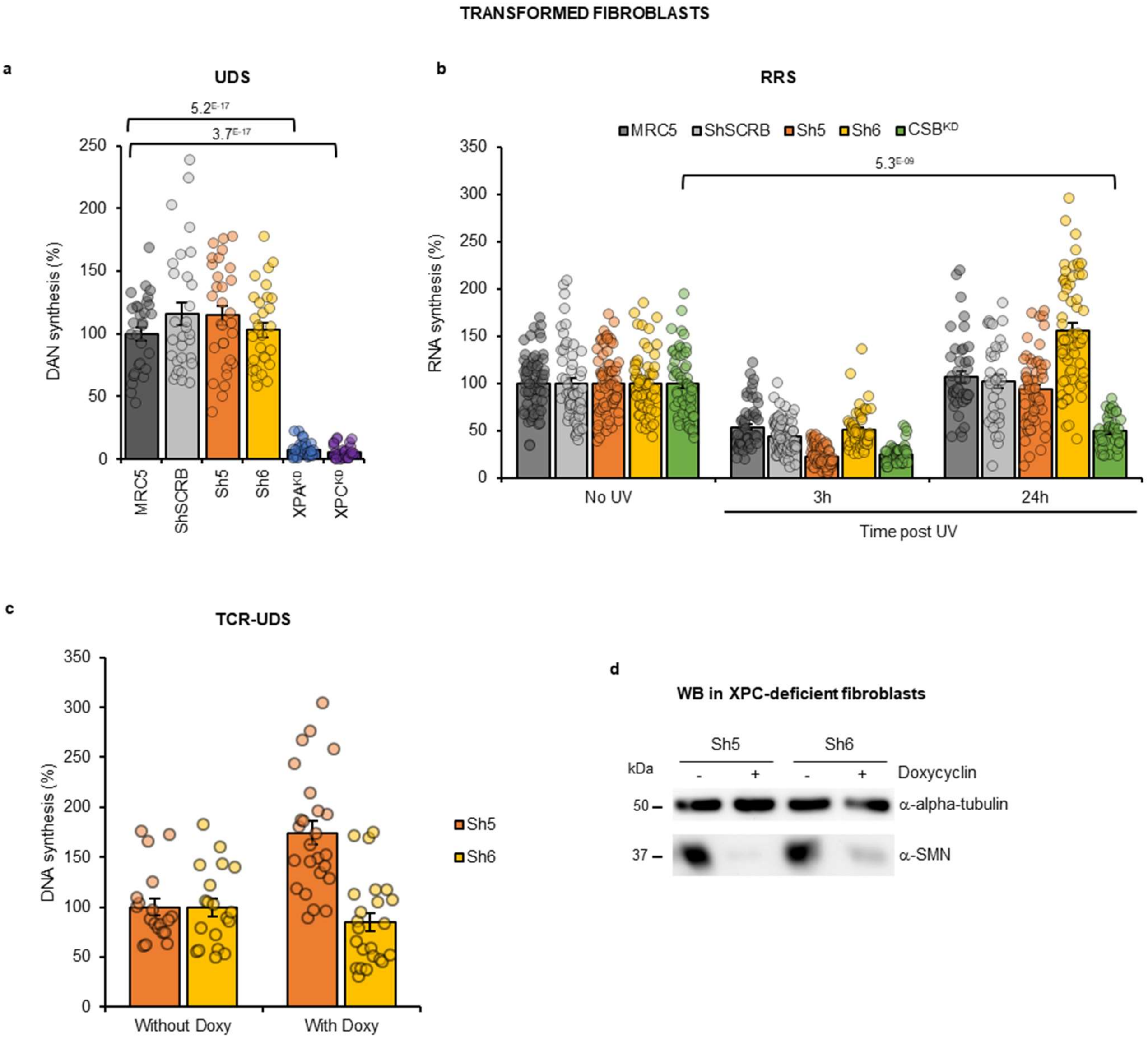

Figure S4: SMN cells are proficient in NER

a. Quantification of Unscheduled DNA Synthesis assay (UDS) determined by EdU incorporation after local damage (LD) induction with UV-C (100J/m²) in transformed fibroblasts. Data are represented as mean values +/- SEM. At least 30 LDs was quantified from one representative experiment. The p-value correspond to a student's test with two-tailed distribution and two-sample unequal variance to compare with MRC5 condition. **b.** Quantification of RNA Recovery Synthesis (RRS) assay determined by EU incorporation after UV-C (10J/m²) exposure in transformed fibroblasts. Data are represented as mean values +/- SEM. At least 35 cells was quantified from one representative experiment. The p-value correspond to a student's test with two-tailed distribution and two-sample unequal variance to compare after irradiation with No UV condition.. **c.** Quantification of TCR-UDS assay determined by EdU incorporation after LD induction with UV-C (100J/m²) in GG-NER deficient cells (XPC-/- cells) expressing or not Sh5-SMN or Sh6-SMN. Data are represented as mean values +/- SEM. At least 19 LDs was quantified from one representative experiment. The p-value correspond to a student's test with two-tailed distribution and two-sample unequal variance to compare with the condition without

Doxycycline. **d.** Western Blot of SMN on whole cell extracts of XPC-deficient cells with sh5-SMN or sh6-SMN. Doxycycline treatment induces the expression of the shRNA against SMN.
Source data of uncropped gel and graphs are provided as a Source Data file.

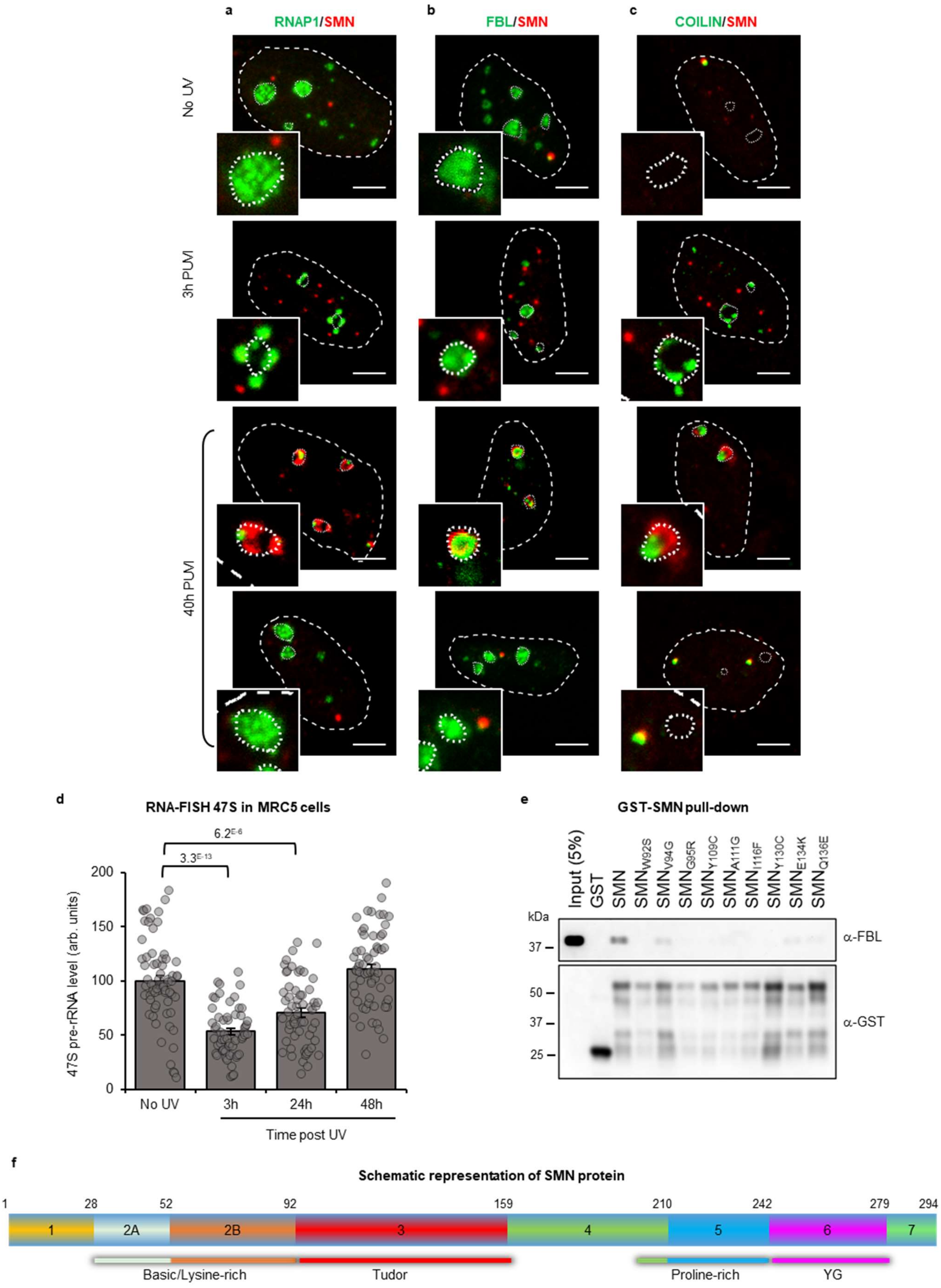

Figure S5: SMN and its partners during DNA repair

Representative microscopy images of immunofluorescence (IF) assay in MRC5 cells showing, after 16J/m² UV-C irradiation, the localization of SMN (red) and **a.** RNAP1, **b.** FBL or **c.** Coilin (green) at different times Post UV-Irradiation (PUVI). Nuclei and nucleoli are indicated by dotted lines. Scale bar: 5 µm. **d.** Quantification of RNA-FISH assay showing the 47S pre-rRNA level in MRC5-SV cells after 3h, 24h and 48h of UV-C exposure (16J/m²). Data are represented as mean values +/- SEM. At least 55 cells was quantified from one representative experiment. The p-value correspond to a student's test with two-tailed distribution and two-sample unequal variance to compare after irradiation with No UV condition. **e.** GST pull-down assay using purified recombinant SMN protein with different mutations in the Tudor domain found in SMA patients and cellular extracts. **f.** Schematic representation of SMN protein with the different exons as well as domains they encode. The number of amino acids encoded by each exon is indicated.

Source data of uncropped gel and graphs are provided as a Source Data file.

a **KBr03 treatment**

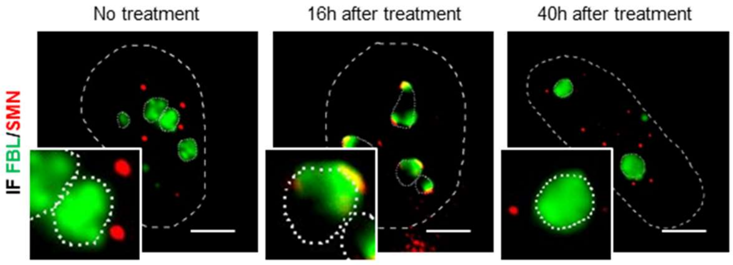

b **Cordycepin experiment**

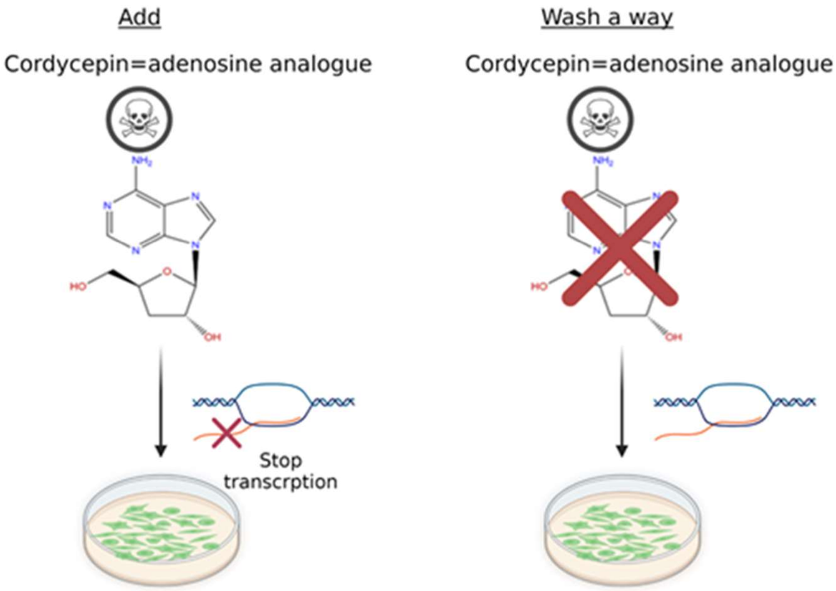

c **Immunofluorescence after Cordycepin treatment**

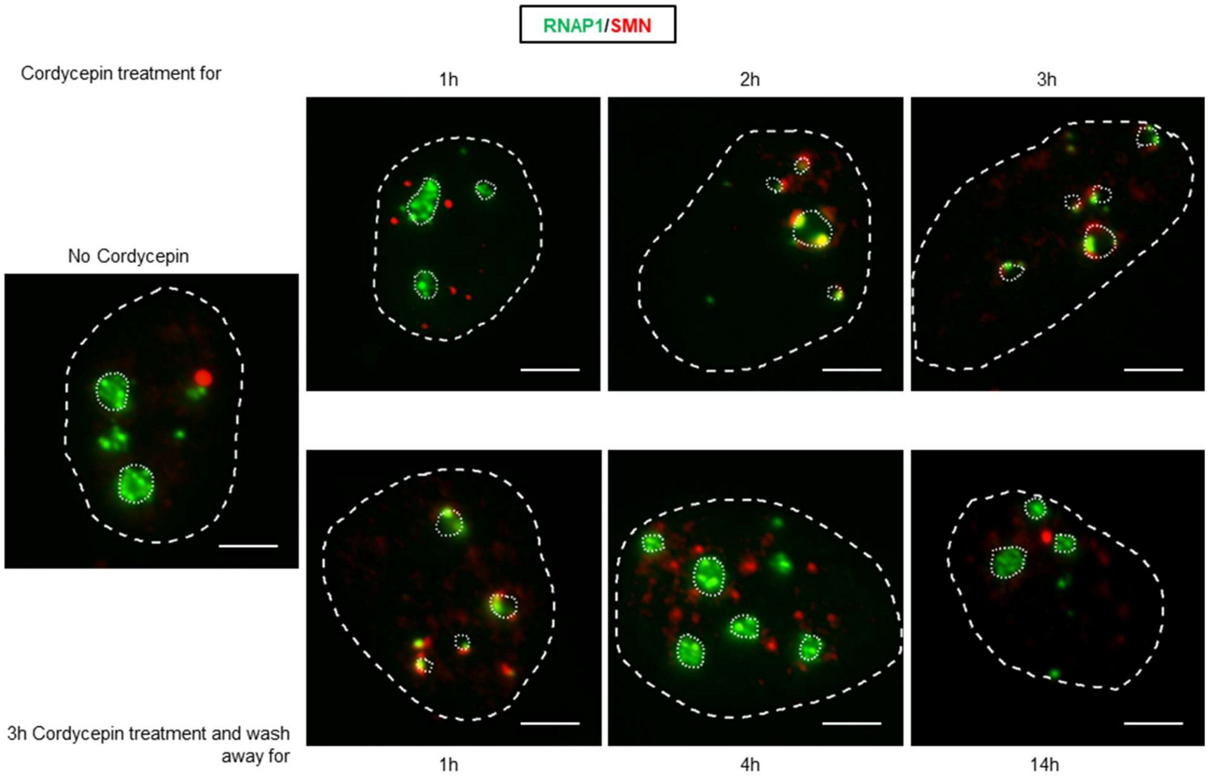

Figure S6: SMN shuttles to the nucleolus after oxidative damage and transcription blockage

a. Representative microscopy images of immunofluorescence (IF) assay against RNAP1 (green) and SMN (red) after 1h of treatment with 80mM $KBrO_3$. Nuclei and nucleoli are indicated by dotted lines. Scale bar: 5µm. **b.** Schematic representation of experiment using Cordycepin. **c.** Representative microscopy images of IF against RNAP1 (green) and SMN (red) after treatment with 50µg/ml of Cordycepin. Nuclei and nucleoli are indicated by dotted lines. Scale bar: 5µm. Source data of uncropped gel and graphs are provided as a Source Data file.

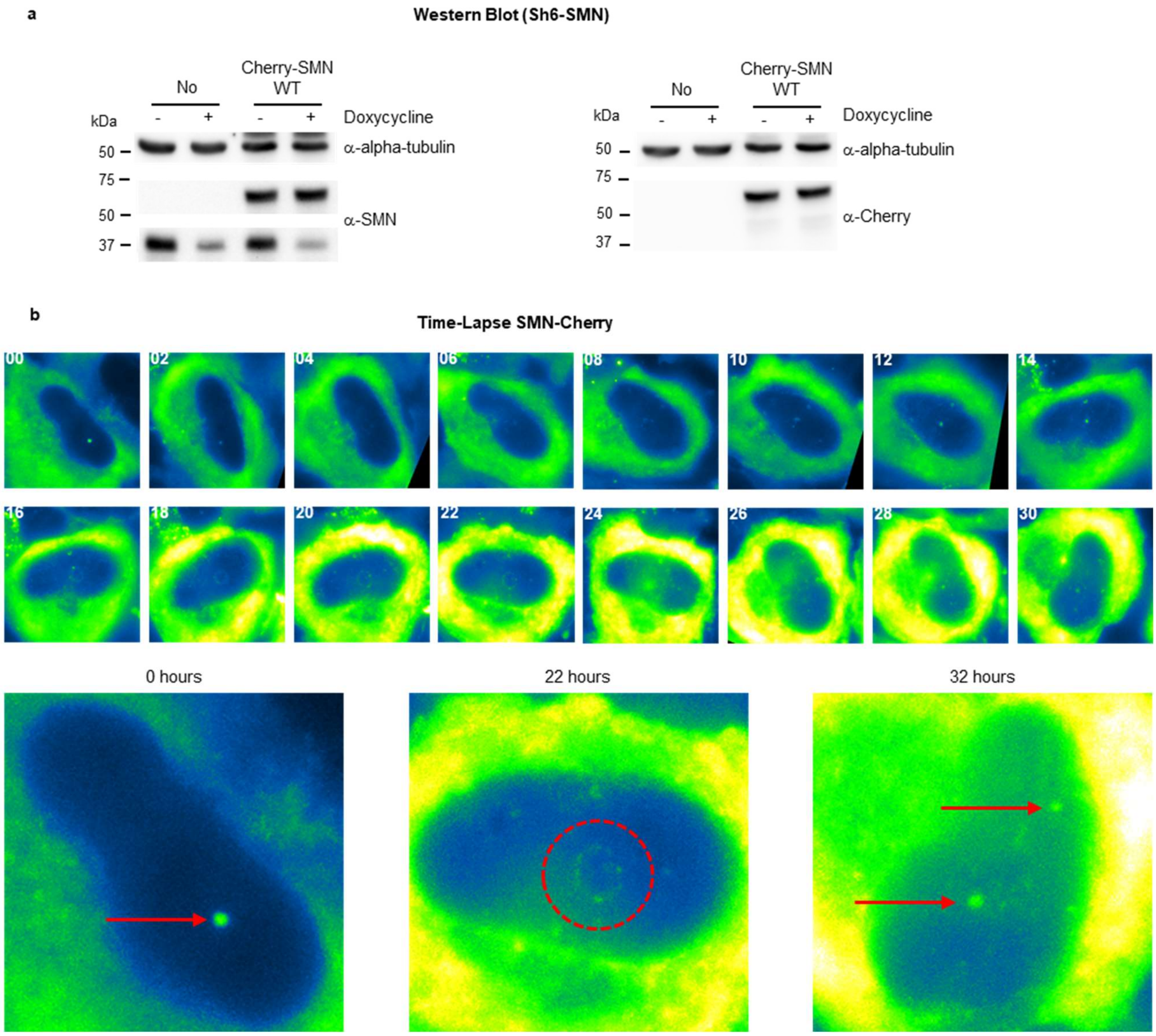

Figure S7: SMN Shuttle to the nucleolus after UV irradiation

a. Western Blot of SMN on whole cell extracts of MRC-SV + Sh6-SMN transfected or not with Cherry-SMN. Doxycycline treatment induce the expression of the ShRNA against SMN endogenous**. b.** Representative image of Cherry-SMN expressing cell, cultured in presence of Doxycycline, irradiated with 16J/m² UV-C and imaged every hour.

Source data of uncropped gel are provided as a Source Data file.

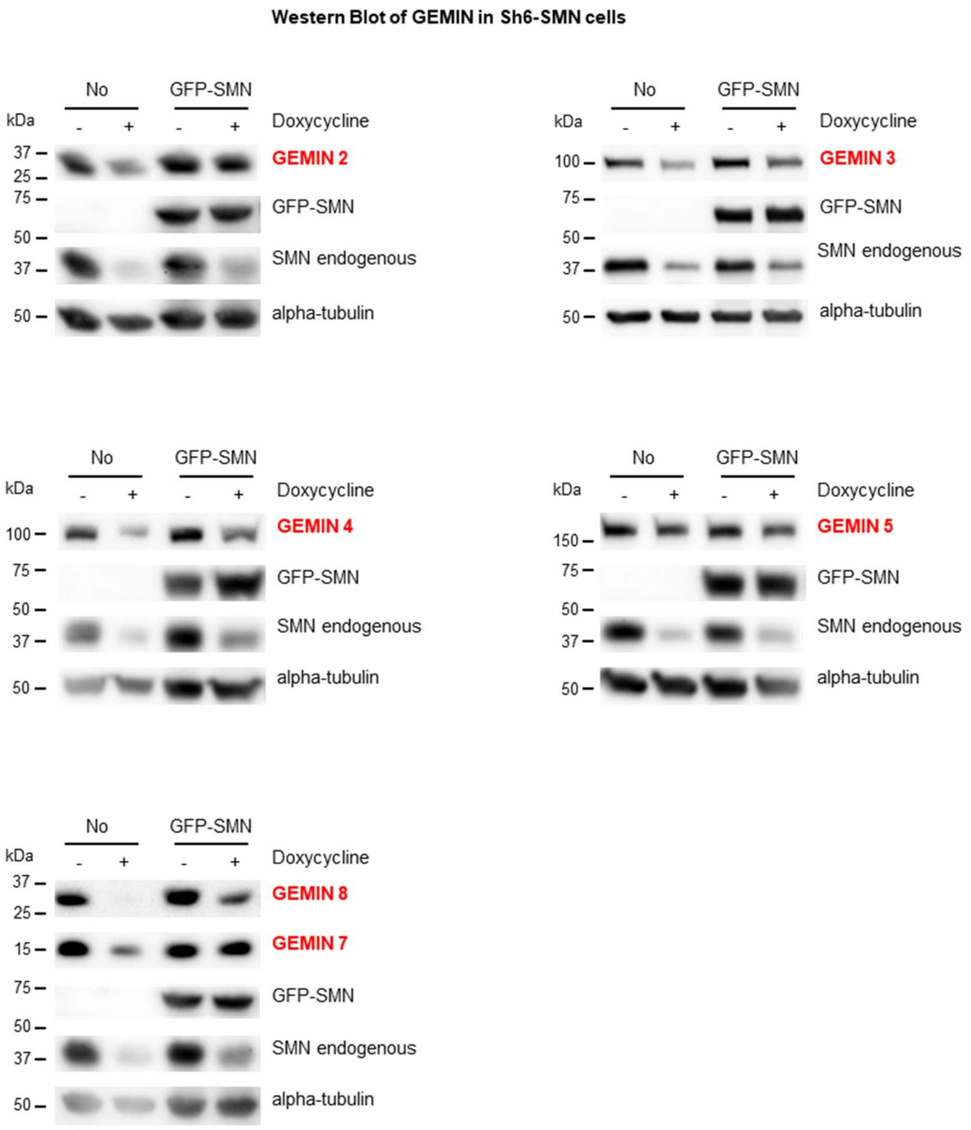

Figure S8: GEMINs level in SMN-depleted cells

Western Blot of GEMIN (2, 3, 4, 5, 7 and 8) on whole cell extracts of MRC-SV + Sh6-SMN transfected or not with GFP-SMN WT. Doxycycline treatment induce the expression of the ShRNA against SMN endogenous.

Source data of uncropped gel are provided as a Source Data file.

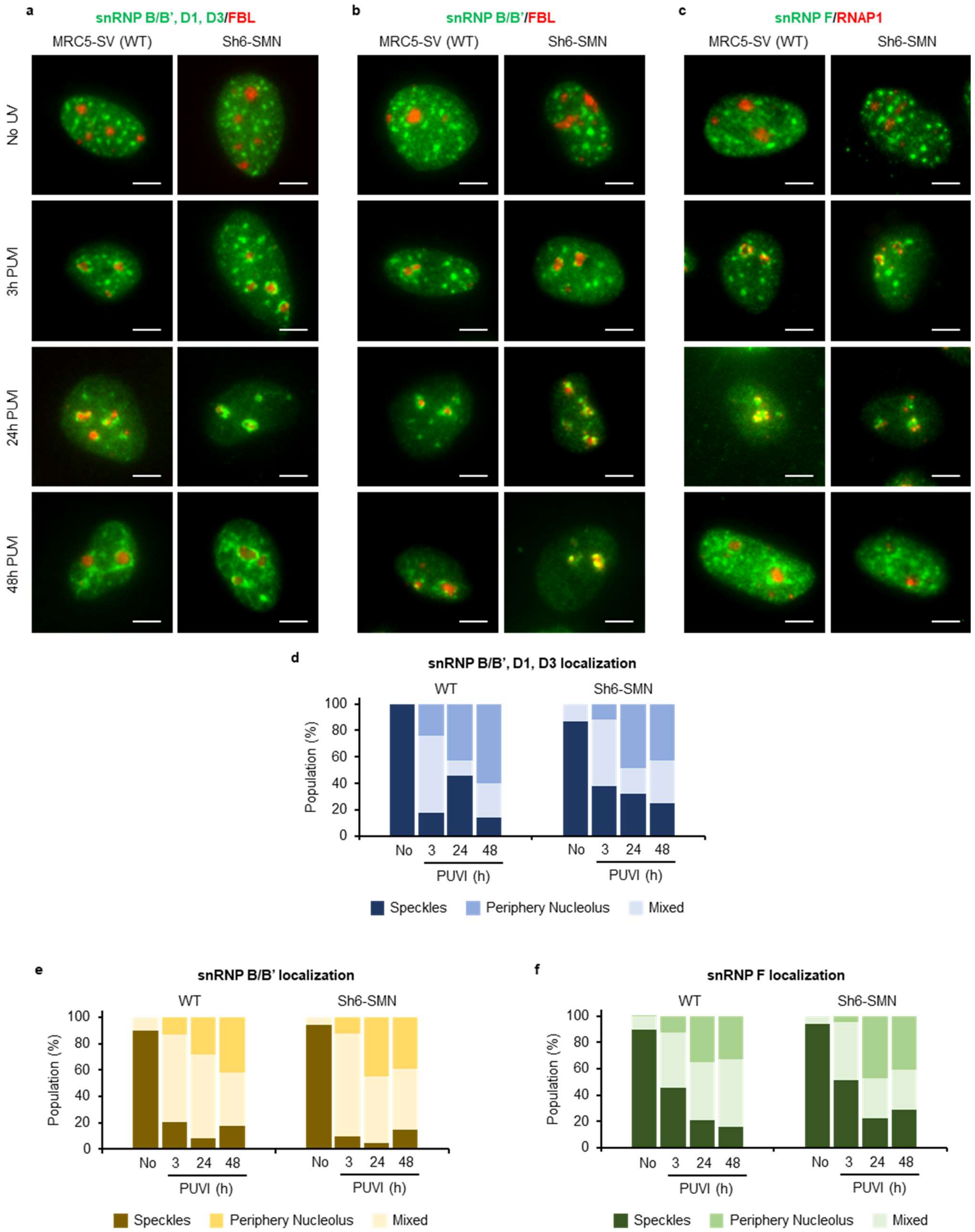

Figure S9: Sm proteins shuttling is SMN-independent

a. b. c. Representative microscopy images of immunofluorescence (IF) assay against **a.** snRNP B/B', D1, D2, **b.** snRNP B/B' and **c.** snRNP F (green) and FBL or RNAP1 (red) in transformed fibroblasts (MRC5 and Sh6-SMN cells), at different

times Post UV-Irradiation (PUVI). Scale bar: 5µm **d. e. f.** Quantification of cells number for localization of **d.** snRNP B/B', D1, D2, **e.** snRNP B/B' and **f.** snRNP F (in Speckles, at the periphery of the nucleolus or mixed localization) at different times post UV-irradiation (PUVI). At least 100 cells from one representative experiment were analyzed.

Source data of graphs are provided as a Source Data file

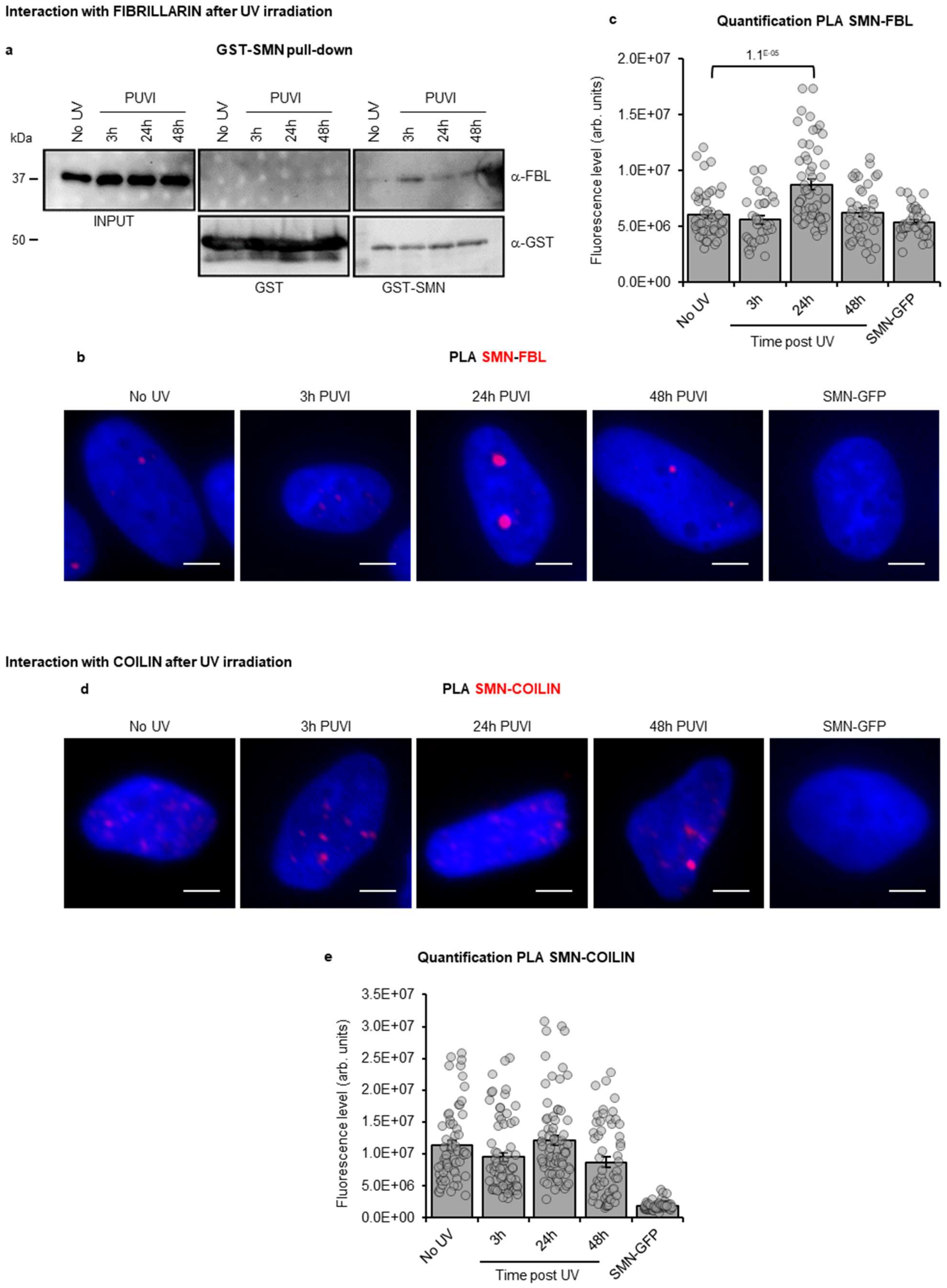

Figure S10: SMN interacts with FBL after UV irradiation.

a. GST pull-down assay using purified recombinant SMN protein and cellular extracts after UV-C irradiation. **b.** Representative microscopy images of proximity ligation assay (PLA) in red showing the interaction between FBL and SMN in WT cells after UV-C irradiation. Scale bar: 5µm. **c.** Quantification of fluorescent signal in the nucleus against the couple SMN-FBL from PLA experiment in WT cells after UV-C irradiation. Data are represented as mean values +/- SEM. At least 30 cells was quantified from one representative experiment. The p-value correspond to a student's test with two-tailed distribution and two-sample unequal variance to compare after irradiation with No UV condition. **d.** Representative microscopy images of PLA In red showing the interaction between COILIN and SMN in WT cells after UV-C irradiation. Scale bar: 5µm. **(E)** Quantification of fluorescent signal in the nucleus against the couple SMN-COILIN from PLA experiment in WT cells after UV-C irradiation. At least 50 cells was quantified from one representative experiment. The p-value correspond to a student's test with two-tailed distribution and two-sample unequal variance to compare after irradiation with No UV condition.

Source data of graphs are provided as a Source Data file.

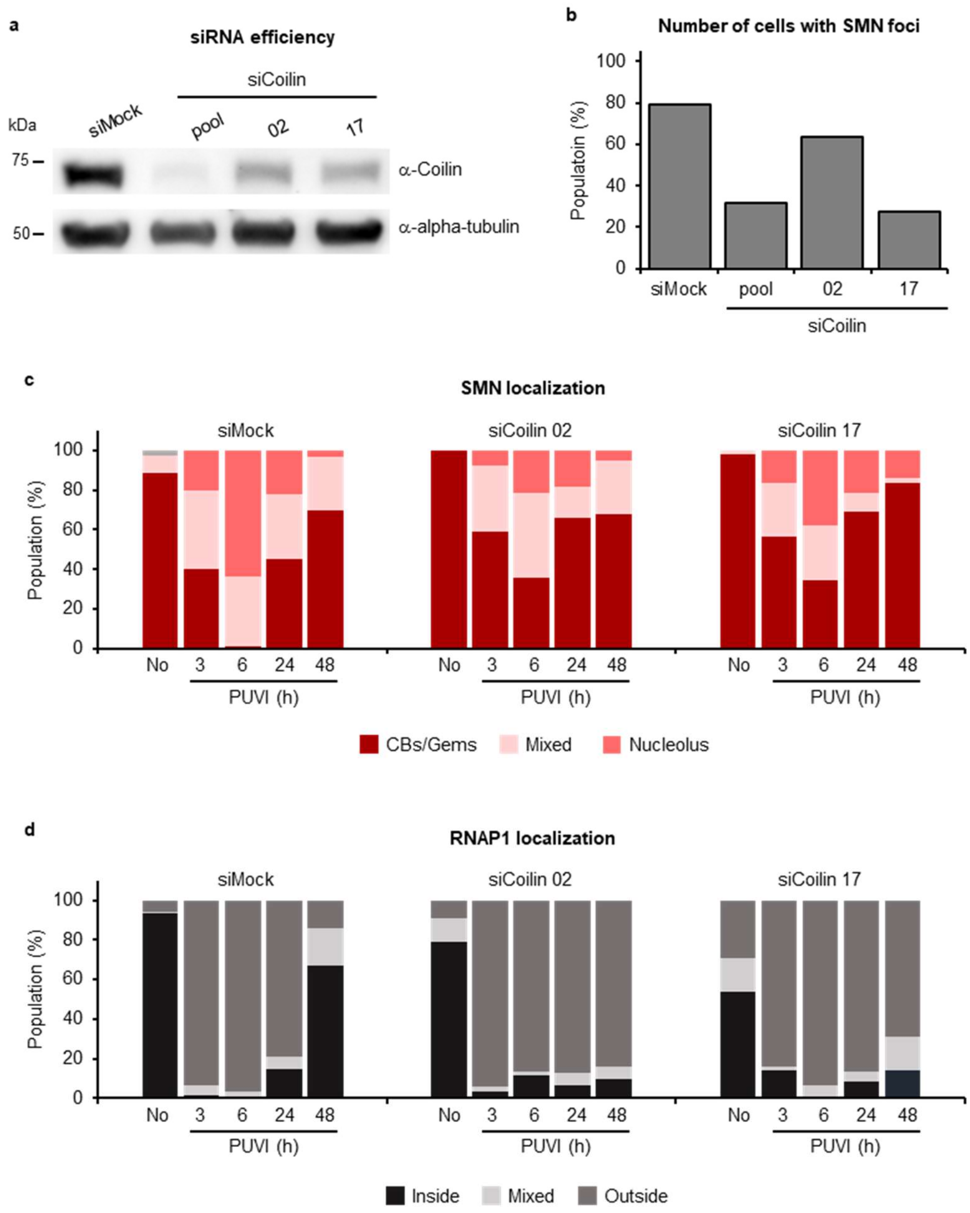

Figure S11: SMN shuttling is Coilin-dependent

a. Western Blot on whole cell extracts of MRC5 cells treated with siCoilin pool or individual. **b.** Quantification of cells number with SMN foci in cells transfected with siMock or siCoilin pool or individual in No UV condition. At least 130 cells from one representative experiment were analyzed. **c. and d.** Quantification of cells number for localization of **c.** SMN (in Cajal Bodies [CBs] or Gems, at the periphery of the nucleolus or mixed localization) and **d.** RNAP1 (inside the nucleolus, outside the nucleolus or mixed localization) at different times post UV-irradiation (PUVI) in cells transfected with siMock or individual siCoilin. At least 50 cells from one representative experiment were analyzed.

Source data of graphs are provided as a Source Data file.

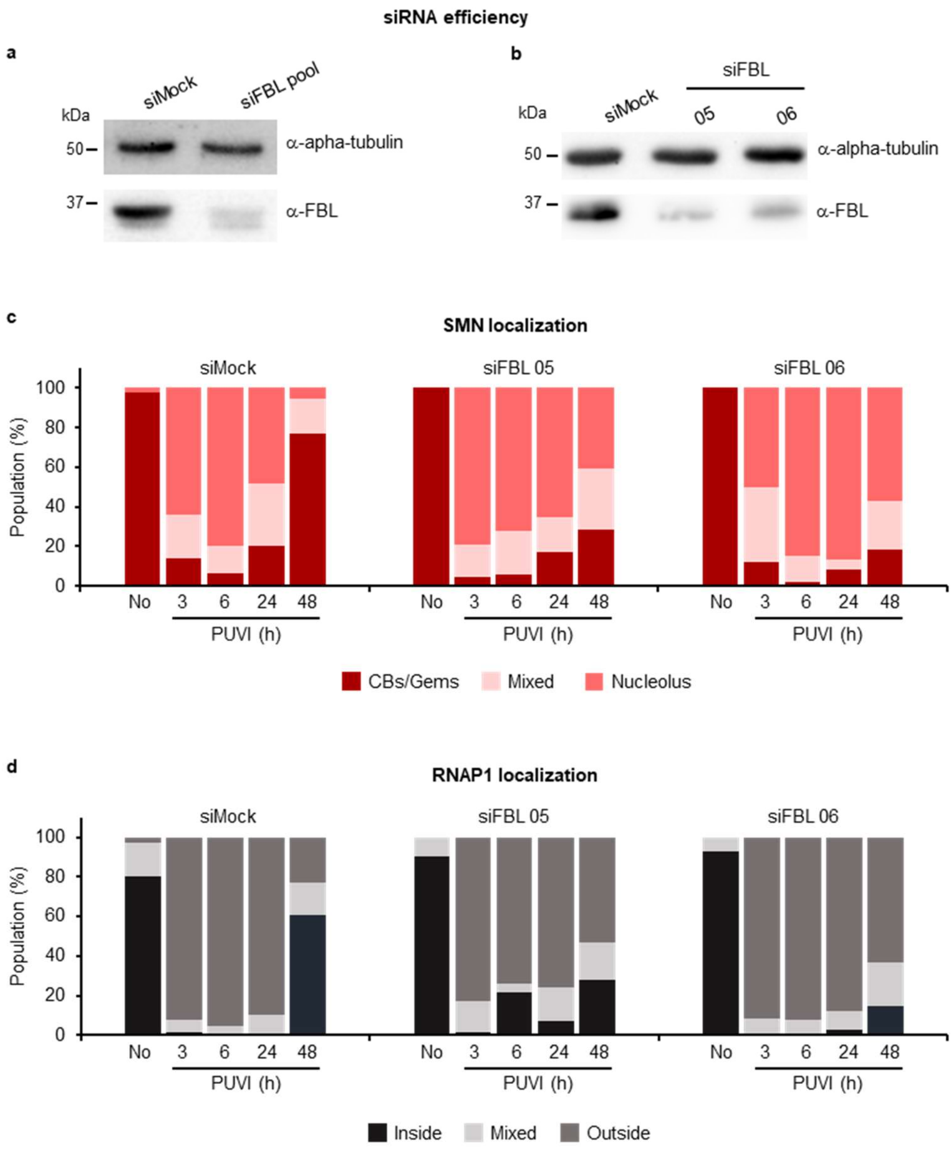

Figure S12: FBL is required for nucleolar rearrangement during DNA repair

a. and b. Western Blot on whole cell extracts of MRC5 cells treated with **a.** siFBL pool or **b.** individual. **c. and d.** Quantification of cells number for localization of **c.** SMN (in Cajal Bodies [CBs] or Gems, at the periphery of the nucleolus or mixed localization) and **d.** RNAP1 (inside the nucleolus, outside the nucleolus or mixed localization) at different times Post UV-Irradiation (PUVI) in cells transfected with siMock or siFBL individual. At least 18 cells from one representative experiment were analyzed.

Source data of graphs are provided as a Source Data file.

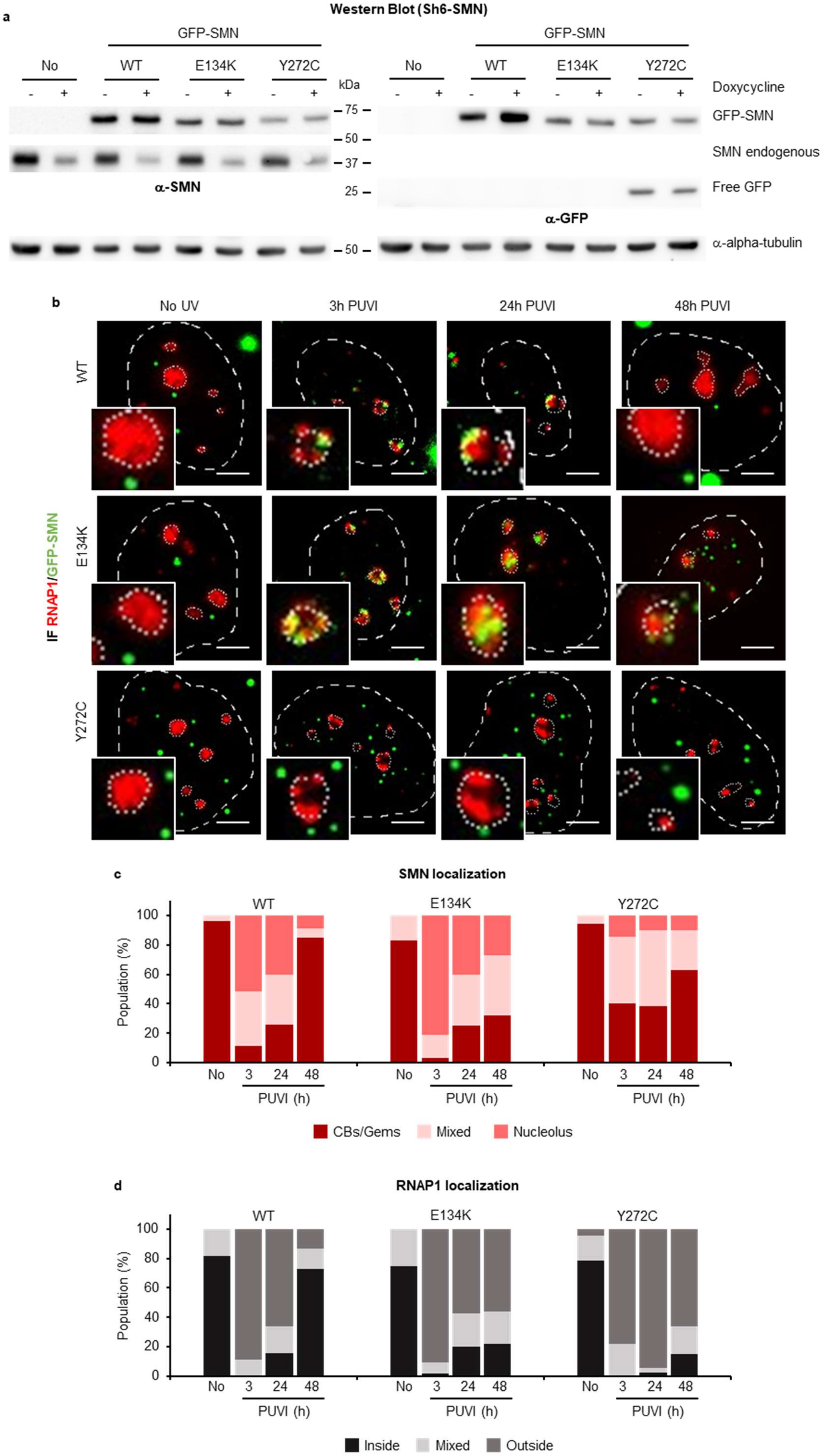
a
Western Blot (Sh6-SMN)
GFP-SMN
No
WT
E134K
Y272C
kDa
Doxycycline
GFP-SMN
SMN endogenous
Free GFP
α-SMN
α-GFP
α-alpha-tubulin
75
50
37
25
b
No UV
3h PUVI
24h PUVI
48h PUVI
IF RNAP1/GFP-SMN
c
SMN localization
Population (%)
PUVI (h)
CBs/Gems
Mixed
Nucleolus
d
RNAP1 localization
Inside
Mixed
Outside

Figure S13: SMN mutants are deficient in nucleolar reorganization during DNA repair.

a. Western Blot on whole cell extracts of MRC5 cells transfected or not with GFP-SMN, GFP-SMN^{E134K} or GFP-SMN^{Y272C}. GFP-SMN is revealed with either SMN (left panel) or GFP (right panel) antibody. Doxycycline treatment induces the expression of the shRNA against SMN endogenous. **b.** Representative microscopy images of immunofluorescence (IF) assay showing the localization of RNAP1 (red) and GFP-SMN (green) in cells depleted of the endogenous SMN, by induction of the sh6-SMN RNA, and expressing GFP-SMN WT (top panel), GFP-SMN^{E134K} (middle panel) or GFP-SMN^{Y272C} (bottom panel). Nuclei and nucleoli are indicated by dotted lines. Scale bar: 5µm. **c. and d.** Quantification of cells number for localization of **c.** SMN (in Cajal Bodies [CBs] or Gems, at the periphery of the nucleolus or mixed localization) and **d.** RNAP1 (inside the nucleolus, outside the nucleolus or mixed localization) at different times PUVI in cells depleted of the endogenous SMN, by induction of the sh6-SMN RNA, and expressing GFP-SMN, GFP-SMN^{E134K} or GFP-SMN^{Y272C}. At least 20 cells from one representative experiment were analyzed.

Source data of graphs are provided as a Source Data file.

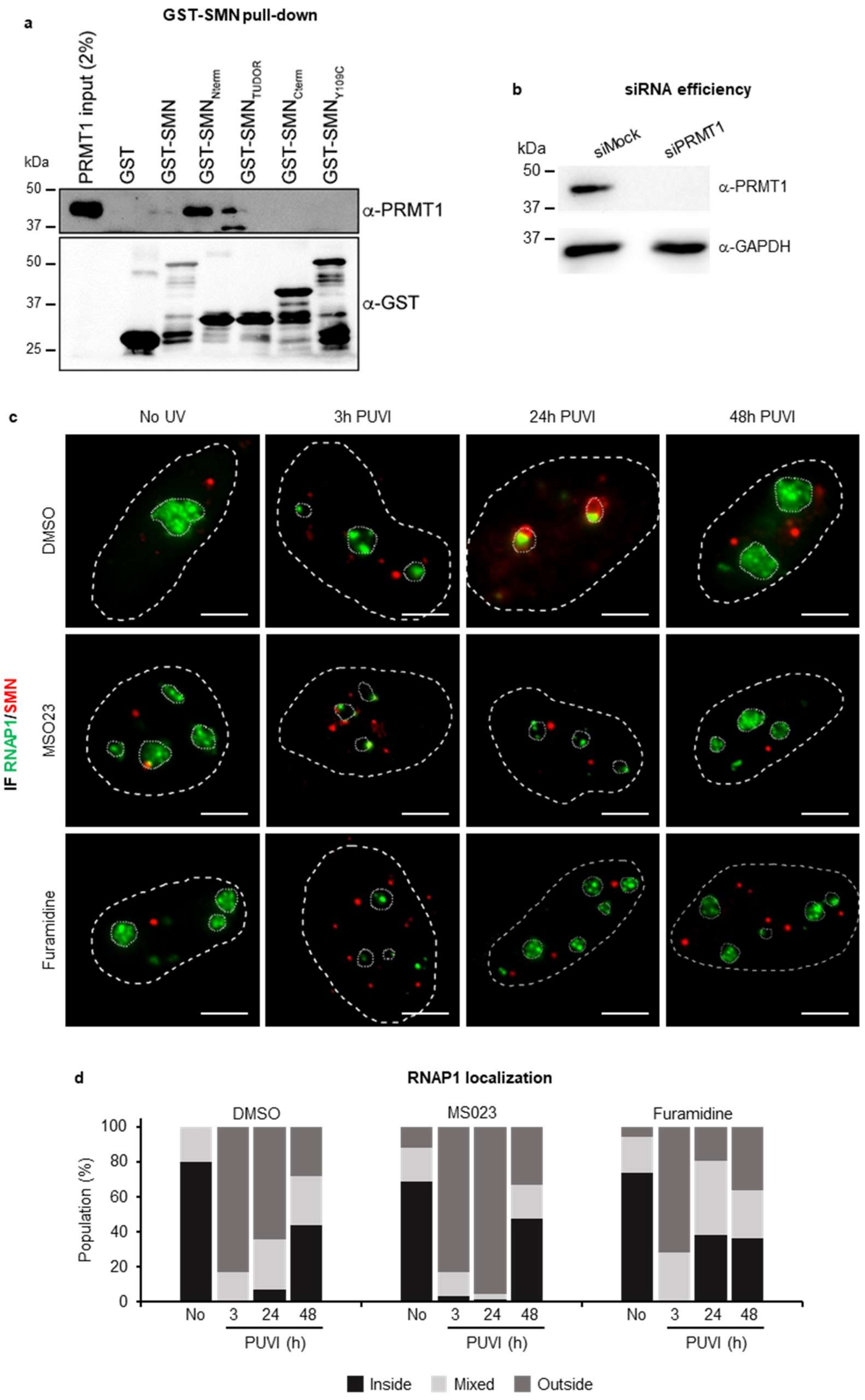

Figure S14: Relation between PRMT1 and SMN.

a. GST pull-down assay using purified recombinant SMN protein with different truncations of the protein and cellular extracts. **b.** Western Blot on whole cell extracts of MRC5 cells treated with siPRMT1. **c.** Representative microscopy images

of immunofluorescence (IF) assay showing the localization of SMN (red) and RNAP1 (green) at different times Post UV-Irradiation (PUVI) in cells in MRC5 cells treated with DMSO, MS023 or Furamidine. Nuclei and nucleoli are indicated by dotted lines. Scale bar: 5µm. **d.** Quantification of cells number for localization of RNAP1 (inside the nucleolus, outside the nucleolus or mixed localization) at different times PUVI in cells treated with DMSO, MS023 or Furamidine. At least 50 cells from one representative experiment were analyzed.

Source data of graphs are provided as a Source Data file.

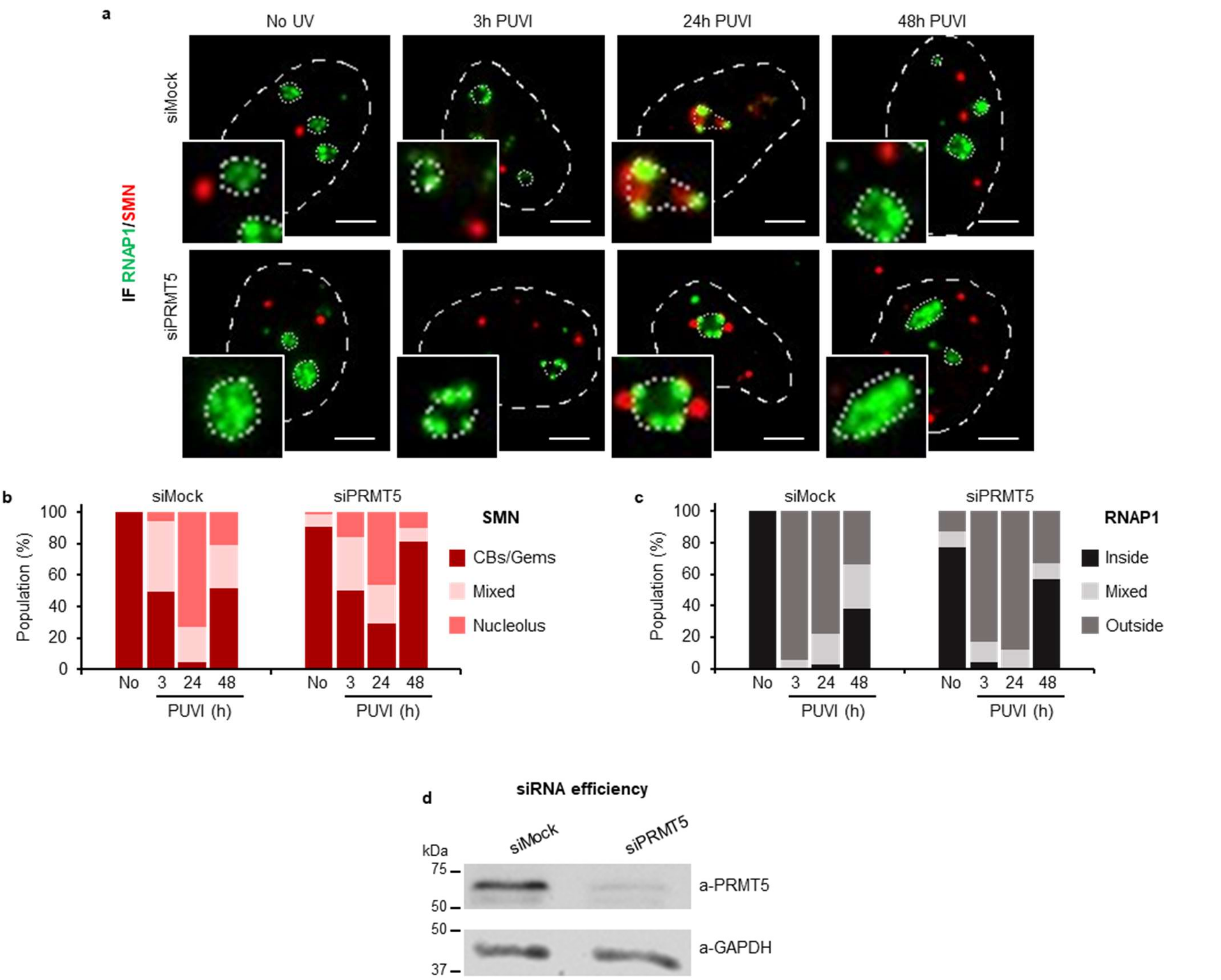

Figure S15: SMN shuttling is PRMT5-independent

a. Representative microscopy images of Immunofluorescence (IF) assay showing the localization of SMN (red) and FBL (green) at different times Post UV-Irradiation (PUVI) in cells transfected with siMock or siPRMT5 pool. Nuclei and nucleoli are indicated by dotted lines. Scale bar: 5µm. **b. and c.** Quantification of cells number for localization of **b.** SMN (in Cajal Bodies [CBs] or Gems, at the periphery of the nucleolus or mixed localization) and **c.** FBL (inside the nucleolus, outside the nucleolus or mixed localization) at different times PUVI in cells transfected with siMock or siPRMT5 pool. At least 50 cells from one representative experiment were analyzed. **d.** Western Blot on whole cell extracts of MRC5 cells treated with siPRMT5.

Source data of graphs are provided as a Source Data file.

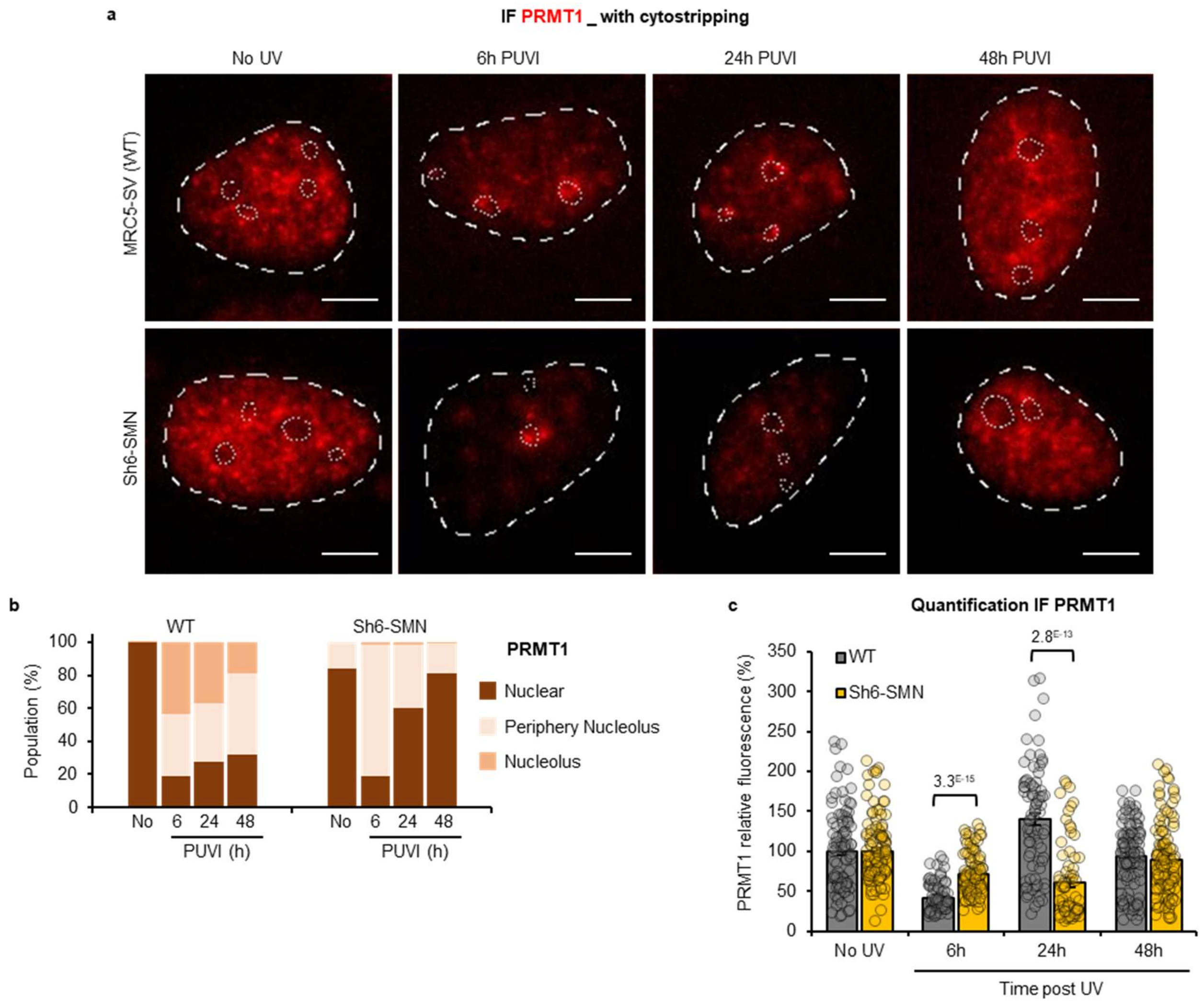

Figure S16: PRMT1 localization and expression after UV irradiation in the presence or absence of SMN

a. Representative microscopy images of Immunofluorescence (IF) assay with cytostripping showing the localization of PRMT1 (red) in MRC5 and Sh6-SMN cells at different times Post UV-Irradiation (PUVI). Nuclei and nucleoli are indicated by dotted lines. Scale bar: 5µm. **b.** Quantification of cells number for localization of PRMT1 (pan nuclear, at the periphery of the nucleolus or inside the nucleolus) at different times PUVI in WT or SMN depleted cells (Sh6-SMN). At least 185 cells from one representative experiment were analyzed. **c.** Quantification of fluorescent signal in the nucleus from the IF with cytostripping against PRMT1. Data are represented as mean values +/- SEM. At least 65 cells was quantified from one representative experiment. The p-value correspond to a student's test with two-tailed distribution and two-sample unequal variance to compare sh6-SMN with WT.

Source data of graphs are provided as a Source Data file.

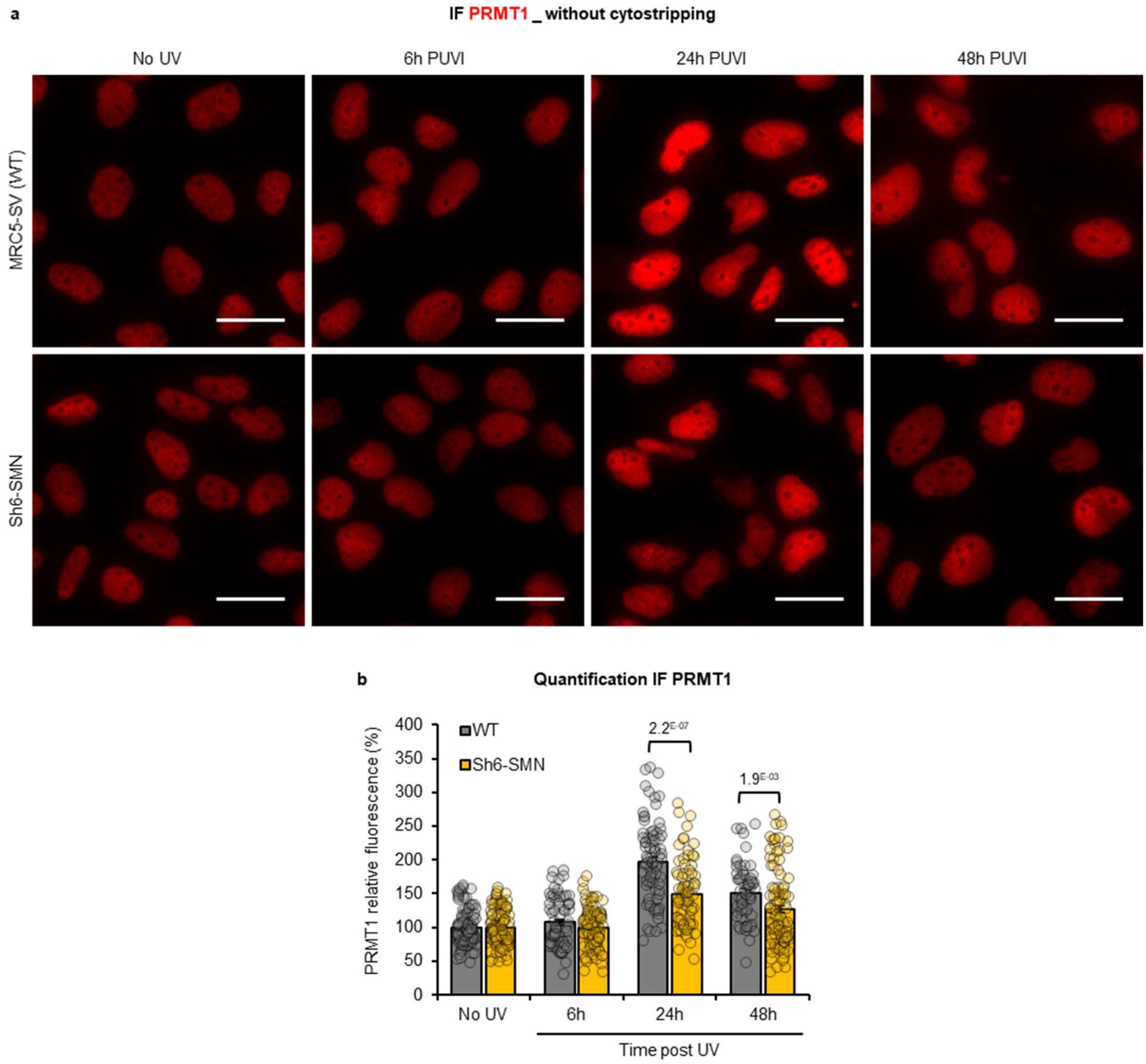

Figure S17: PRMT1 localization and expression after UV irradiation in the presence or absence of SMN (without cytostripping)

a. Representative microscopy images of Immunofluorescence (IF) without cytostripping showing the localization of PRMT1 (red) in MRC5 and Sh6-SMN cells at different times Post UV-Irradiation (PUVI). Nuclei and nucleoli are indicated by dotted lines. Scale bar: 5µm. **b.** Quantification of fluorescent signal in the nucleus from the IF without cytostripping against PRMT1. Data are represented as mean values +/- SEM. At least 60 cells was quantified from one representative experiment. The p-value correspond to a student's test with two-tailed distribution and two-sample unequal variance to compare sh6-SMN with WT.

Source data of graphs are provided as a Source Data file.

MATERIALS AND METHODS supplemental

Cells culture

Primary fibroblast cells from unaffected (C5RO [RRID: CVCL_ZP35]) and CSB-deficient patients (CS1AN; [RRID:CVCL_L471]) come from Erasmus MC in Rotterdam and were cultured in DMEM supplemented with 10% FBS and 1% P/S. SMA type I patients (GM00232 [RRID: CVCL_Y965]) fibroblast cell lines were obtained from Coriell Cell Repositories and cultured in MEM supplemented with 15% non-inactivated FBS, 1% non-essential amino acids and 1% P/S. All primary fibroblasts are incubated at 37°C with 3% O_2 and 5% CO_2. All cell lines are regularly tested negative for mycoplasma contamination.

Human iPSC-derived motoneurons (hMNs) were generated as previously described by [1] Human iPSCs were dissociated with TrypLE (Gibco) and resuspended in motoneuron medium (MNB) containing DMEM-F12 Glutamax/Neurobasal (1:1 ratio; Gibco), N2 supplement (Gibco), B27 without vitamin A supplement (Gibco), β-mercaptoethanol (0,1%; Gibco), Penicillin/Streptomycin (0,1%; Gibco), supplemented with small molecules including ascorbic acid (0.5 µM; Sigma-Aldrich), SB431542 (20 µM; TOCRIS-BioTechne, Minneapolis, MN, USA), LDN193189 (0.2 µM; Miltenyi Biotec), CHIR99021 (3 µM; Miltenyi Biotec) and Y-27632 (10 µM; STEMCELLS Technologies, Vancouver, Canada) for the first day of differentiation. Cells were seeded in suspension into 6 well plate (Dutscher, Bernolsheim, France) treated with anti-adherence rinsing solution (Stemcell, Technologies) to form spheroids. During the entire culture process, small molecules were added at different time points including retinoic acid (0.1 µM RA; Sigma-Aldrich), Smoothened Agonist (0.5 µM SAG; STEMCELLS Technologies), Brain-Derived Neurotrophic Factor (10 ng/mL BDNF; PreproTech, Rocky Hill, NJ, USA) and γ-secretase inhibitor (10 µM DAPT; STEMCELLS Technologies). Then, spheroids were dissociated at DIV 10 (days in vitro) with trypLE to obtained hMNs progenitors that were plate on glass coverslips coated with poly-L-ornithine (Sigma-Aldrich) and laminin at 1-2µg/ml (Sigma-Aldrich) or dispensed into cryovials and freezed using CryoMed Controlled-Rate Freezer (Thermo Fisher Scientific). hMNs were maintain in culture for maturation in MNB supplemented with BDNF and GDNF.

Construction and expression of GFP-SMN and Cherry-SMN fusion protein

The GFP-SMN WT and GFP-SMN E134K were previously described in [2]. The GFP-SMN Y272C was generated using standard PCR-based site-directed mutagenesis. For mCherry-SMN WT, SMN cDNA was inserted in mCherry-N1.

Cherry-SMN WT and GFP-SMN WT, E134K and Y272C stably expressing cell lines were produced by transfecting the plasmid in MRC5-SVcells containing sh6-SMN. FuGENE 6 Transfection Reagent (Promega) was used according to the manufacturer' protocol. The selection was performed with G418 at 200 µg/mL.

Treatment

RNAP1 transcription inhibition has been achieved by incubation in medium containing Cordycepin at 50µg/mL. Resumption of transcription has been obtained by replacement of Cordycepin medium with normal medium after two washes with PBS. No treated cells were used as control.

The stock of KBro3 is freshly diluted at 80mM in warm DMEM. After one wash with PBS, cells are incubated one hour with KBro3. After this incubation, cells were washed two time with PBS and incubated with medium at 37°C with 5% Co2 for different period of time.

Transfection of small interfering RNAs (siRNAs)

The small interfering RNA (siRNAs) used in this study are: siPRMT5, GGCCAUCUAUAAAUGUCUG (10mM), SiCOILIN #02, GAGAGAACCUGGGAAAUU (10nM), SiCOILIN 17, CGGAGUGUGCUGCGGGUUU (10nM), siFBL 05 GGUCGAGGCGGAGGCUUUA (10nM), siFBL 06, AAUGGUGGAUGUGAUCUUU (10nM).The final concentration used for each siRNA is indicated in parentheses.

GST-SMN purification and GST pull-downs

SMN (full-length or truncated) cDNA was cloned in pGEX6P1 between *BamHI* and *XhoI* sites and transformed in BL21 (DE3) cells (200131; Agilent). Single colonies were grown overnight in 2.5 mL LB broth, scaled up to 250 mL, grown at 37 °C until density at OD_{600} reached 0.6, then GST or GST-SMN were induced with 0.2 mM IPTG overnight. The next day, cells were collected by centrifugation and resuspended in 10 mL lysis buffer (50mM Tris pH 8.0, 150mM NaCl, 0.05% NP40, supplemented with PIC). While working on ice, cells were briefly sonicated and extracts clarified by centrifugation. Recombinant proteins were then purified using Glutathione-sepharose beads (tumbling at 4 °C overnight), washed extensively with lysis buffer, and released from the beads using elution buffer (100mM Tris pH 8.0, 10% Glycerol, 15mg/mL reduced glutathione).

GST pull-downs were performed in 600 µL TAP buffer (50mM Tris pH 7.5, 200mM NaCl, 0.1% Triton-X100, and 10% glycerol supplemented with PIC) with 5µg GST and 85µL HEK293T whole cell lysate (1 x 100 mm plate lysed in 1 mL TAP buffer). A 10% input (8.5µL in 20µL Laemmli sample buffer) was set aside. Samples were incubated 2-3h at 4°C with rotation, then 25µL Glutathione-sepharose beads were added for 1h. Finally, the beads were washed 4 times with 1mL TAP buffer and finally resuspended in 20µL Laemmli sample buffer before immunoblotting analyses.

For time course experiments following UV-induced DNA damage, MRC5-SV cells were lysed in Pierce IP lysis buffer (Thermo 87787) and 17µg of proteins (amount available per pull-down) were used as above.

Recovery of RNA synthesis (RRS) assay

Cells were grown on coverslips. RNA detection was done using a Click-iT RNA Alexa Fluor Imaging kit (Invitrogen, C10330), according to the manufacturer's instructions. Briefly, cells were UV-C irradiated (10 J/m^2) and incubated for 3 or 24 h at 37°C. Then, cells were incubated for 2 hours with 5-ethynyl uridine (EU). After fixation and permeabilization, cells were incubated for 30min with the Click-iT reaction cocktail containing Alexa Fluor Azide 594. After washing, the coverslips were mounted with Vectashield (Vector). The average fluorescence intensity per nucleus was estimated after

background subtraction using ImageJ and normalized to not treated cells. At least 50 cells were images for each condition of each cell lines.

Unscheduled DNA synthesis (UDS or TCR-UDS).

Cells were grown on coverslips. After local irradiation, cells were incubated for 3 or 8 hours (UDS and TCR-UDS respectively) with 20µM of 5-ethynyl-2'-deoxyuridine (EdU), fixed with 4% PFA for 15min at 37°C and permeabilized with PBS and 0.5% Triton X-100 for 20min. Then, cells were blocked with PBS+ for 30min and subsequently incubated for 1h at RT with mouse monoclonal anti-yH2AX antibody (Ser139 [Upstate, clone JBW301]) 1:500 diluted in PBS+. After extensive washes with PBS containing 0.5% Triton X100, cells were incubated for 45min at RT with secondary antibodies conjugated with Alexa Fluor 594. Next, cells were washed several times and then incubated for 30 min with the Click-iT reaction cocktail containing Alexa Fluor Azide 488 (Invitrogen, C10337). After washing, the coverslips were mounted with Vectashield containing DAPI (Vector). Images of the cells were obtained with the same microscopy system and constant acquisition parameters.

Images were analyzed as follows using ImageJ and a circle of constant size for all images: (i) the background signal was estimated in the nucleus (avoiding the damage, nucleoli and other non-specific signal) and subtracted, (ii) the locally damaged area was defined by using the yH2AX staining, (iii) the average fluorescence correlated to the EdU incorporation was then measured and thus an estimate of DNA synthesis after repair was obtained. For UDS experiment, at least 30 cells were imaged for each condition. For TCR-UDS, at least 20 cells were imaged for each condition.

Primary Antibodies

The following primary antibodies were used:

Antibody against	Manufacturer	Catalog Nr	Source	IF	WB
Alpha-Tubulin	Sigma-aldrich	T6074	Mouse		1/50 000
Cherry	Abcam	Ab167453	Rabbit		1/2000
Coilin	Proteintech	10967-1-AP	Rabbit	1/500	1/5000
CSB	Santa Cruz Biotechnology	sc398022	Mouse		1/100
Fibrillarin (FBL)	Abcam	ab5821	Rabbit	1/500	1/500
GAPDH	Abcam	Ab9484	Mouse		1/1000
GEMIN2	ABclonal	A3082	Rabbit	1/500	
GEMIN2	Santa Cruz Biotechnology	Sc166162	Mouse		1/1000
GEMIN3	Santa Cruz Biotechnology	sc374373	Mouse	1/1000	1/100
GEMIN4	Santa Cruz Biotechnology	sc365424	Mouse	1/1000	1/250
GEMIN5	Proteintech	24897-1-AP	Rabbit	1/500	1/2000
GEMIN7	Proteintech	10625-1-AP	Rabbit		1/1000

GEMIN8	Santa Cruz Biotechnology	Sc130669	Rabbit		1/1000
GFP	Sigma-aldrich	SAB4701015	Rabbit		1/1000
GST	Abcam	ab3416	Rabbit		1/3000
PRMT1	Abcam	ab190892	Rabbit	1/5000	1/1000
PRMT5	Upstate	07-405	Rabbit		1/1000
RNAP1	Santa Cruz Biotechnology	sc48385	Mouse	1/500	
SMN	Proteintech	11708-1-AP	Rabbit	1/100	
SMN	BD Biosciences	610646	Mouse	1/500	1/5000
Y12 (snRNP B/B, D1, D3)	NeoMarkers	MS-450-PO	Mouse	1/1000	
snRNPB/B'	Santa Cruz Biotechnology	Sc374009	Mouse	1/500	
snRNPF	Proteintech	14977-1-AP	Rabbit	1/1000	

REFERENCES

1. Maury, Y. *et al.* Combinatorial analysis of developmental cues efficiently converts human pluripotent stem cells into multiple neuronal subtypes. *Nat. Biotechnol.* **33**, 89–96 (2015).

2. Sanchez, G. *et al.* A novel function for the survival motoneuron protein as a translational regulator. *Hum. Mol. Genet.* **22**, 668–684 (2013).
